# Altered Osteogenic Differentiation in Mesenchymal Stem Cells Isolated from Compact Bone of Chicken Treated with Varying Doses of Lipopolysaccharides

**DOI:** 10.3390/biom13111626

**Published:** 2023-11-07

**Authors:** Venkata Sesha Reddy Choppa, Guanchen Liu, Yuguo Hou Tompkins, Woo Kyun Kim

**Affiliations:** Department of Poultry Science, University of Georgia, Athens, GA 30605, USA; venkataseshareddy.choppa@uga.edu (V.S.R.C.); guanchen.liu@uga.edu (G.L.); yuguot@uga.edu (Y.H.T.)

**Keywords:** broilers, bone development, interleukin 1 beta, lipopolysaccharides, osteogenic differentiation

## Abstract

Persistent inflammation biologically alters signaling molecules and ultimately affects osteogenic differentiation, including in modern-day broilers with unique physiology. Lipopolysaccharides (LPS) are Gram-negative bacterial components that activate cells via transmembrane receptor activation and other molecules. Previous studies have shown several pathways associated with osteogenic inductive ability, but the pathway has yet to be deciphered, and data related to its dose-dependent effect are limited. Primary mesenchymal stem cells (MSCs) were isolated from the bones of day-old broiler chickens, and the current study focused on the dose-dependent variation (3.125 micrograms/mL to 50 micrograms/mL) in osteogenic differentiation and the associated biomarkers in primary MSCs. The doses in this study were determined using a cell viability (MTT) assay. The study revealed that osteogenic differentiation varied with dose, and the cells exposed to higher doses of LPS were viable but lacked differentiating ability. However, this effect became transient with lower doses, and this phenotypic character was observed with differential staining methods like Alizarin Red, Von Kossa, and alkaline phosphatase. The data from this study revealed that LPS at varying doses had a varying effect on osteogenic differentiation via several pathways acting simultaneously during bone development.

## 1. Introduction

**Figure 1:**
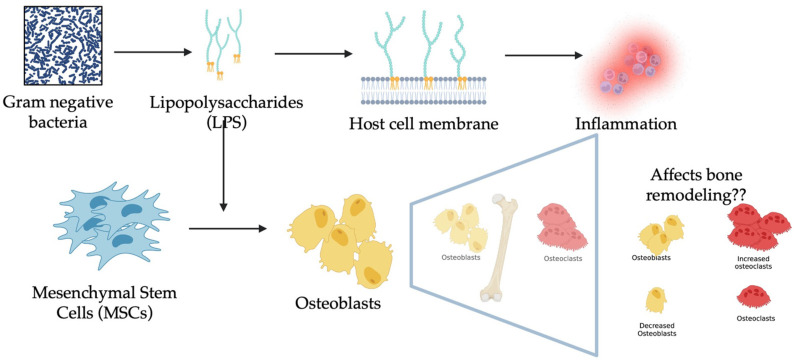
Illustrates the objective of the current study representing the source of LPS, along with their effect, hypothesized to establish an in vitro model for understanding inflammatory bone diseases.

The genetic potential of chicken has increased with the need for global production of chicken meat, making chickens more prone to skeletal disorders [1,2,3]. These issues can only be alleviated using novel methods which can be achieved using studies on mesenchymal stem cells (MSCs) to provide crucial insights into optimal skeletal development [4]. This can be achieved by exploiting the ability of MSCs to study mechanisms like tissue repair, homing potential to understand the biology, as well as defining therapeutic targets [5,6]. Bone MSCs have a strong self-renewing ability and multilineage differentiation potential with an important role in bone turnover and metabolism. In contrast, this is affected by acute or chronic inflammatory mediators such as lipopolysaccharides (LPS). LPS are Gram-negative bacterial components that stimulate the immune system, leading to systemic inflammation. These are usually released into circulation during dysbiosis and abundant Gram-negative bacterial proliferation, which leads to strong inflammatory reactions even at low concentrations, inducing sepsis in the host when translocated [7]. On the other hand, LPS activate a TLR4-MD2 complex, which further activates the NF-κB pathway, ultimately leading to the production of cytokines and chemokines. Additionally, intracellular LPS can trigger cytokine release via LPS binding protein (LBP), which is independent of the former [8,9]. Studies on osteogenic potential are limited to none in chicken isolated MSCs, but human MSCs studies have revealed that TLR4 activation by LPS activates NF-κB and increases proliferation and osteogenic differentiation [10]. In congruity, similar studies have shown the effect of inflammatory mediators on the proliferation, migration, and differentiation of MSCs [11]. In contrast, toll-like receptor (TLR) 4 generates osteoclast-activating cytokine when triggered by LPS stimulation, as well as having inhibitory effects on osteoblast differentiation [12,13]. Equally, there are other important pathways which are involved in MSC proliferation and differentiation like the Wnt (Wingless Int) pathway and Dicer1 (Endoribonuclease III) signaling [14,15,16]. As a strong activator of the immune system through their release into circulation during septicemia, LPS must be investigated in terms of dose dependency and the prolonged response of MSCs in their osteogenic potential and immunological responses. This study was focused on the effects of varying LPS doses on osteogenic differentiation and the pathways affecting this process. The objective (Figure 1) of this study was to establish an in vitro model for the inflammation associated with bone diseases, besides understanding the pathways involved, which will assist the researchers in the application of the compounds targeting them.

## 2. Materials and Methods

### 2.1. Animal Use and Ethics Statement

The investigation was conducted according to the ARRIVE guidelines, and all experimental procedures and animal utilization were authorized by the Institutional Animal Care and Use Committee at the University of Georgia, Athens, GA, USA (Approval code: A2021 07-025; Approval date: 5 August 2021).

### 2.2. Isolation of MSCs

**Figure 2:**
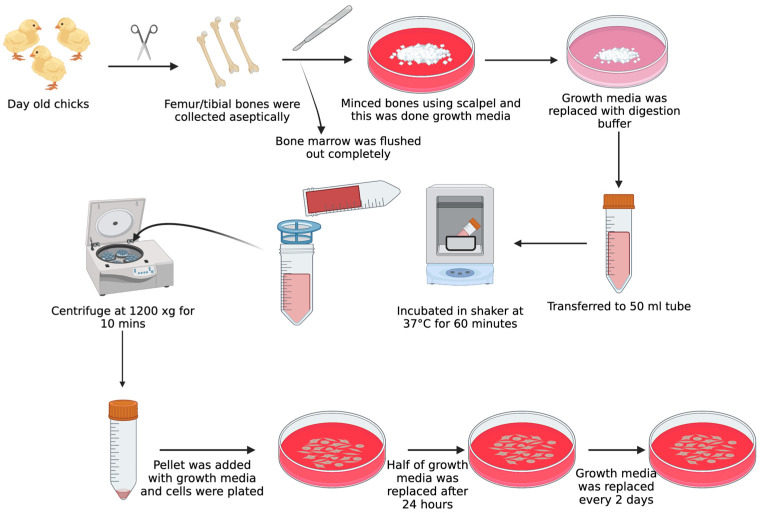
Represents the protocol for isolating MSCs (mesenchymal stem cells) from the compact bones of chicken.

MSC isolation was carried out using previously described methods [17]. After cervical dislocation, the bones of the day-old chicks, femurs, and tibias from both legs, were taken. Following cervical dislocation, the birds were immersed in alcohol for two minutes. The legs were removed from the metacarpal and hip joints. The dissected legs were kept in DMEM (Corning, NY, USA) containing 10% Fetal Bovine Serum (FBS), 100 U/mL penicillin, 100 g/mL streptomycin, and 0.292 mg/mL L-glutamine (Thermo Fisher Scientific, Waltham, MA, USA) until the connective tissues and muscles were completely removed. A scalpel and micro-dissecting scissors were used in a bio-safety cabinet to immediately remove the muscles and connective tissues surrounding the tibias and femurs. The process involved preparing the tibia and femur bones by washing them with a buffer solution containing Phosphate Buffer Saline (PBS) (Corning) and 2% FBS (Hyclone Laboratories Inc., Logan, UT, USA). The bones were then cracked open to remove the bone marrow, which was flushed out with the same buffer solution. The bones were washed again to ensure all bone marrow cells were removed and were then transferred to a cell culture dish containing a digestion medium composed of DMEM, penicillin, streptomycin, collagenase (Sigma-Aldrich, St. Louis, MO, USA), and FBS. The bones were chopped into smaller fragments and digested in a shaking water bath at 37 °C for 60 min. The resulting mixture was filtered, and the bone fragments were rinsed with 10% DMEM before being centrifuged. The supernatant was discarded, and the remaining cells were suspended in 10% DMEM and plated in two 100 mm cell culture dishes. The cells were cultured in a humidified incubator containing 5% CO_2_ (Nuaire, Plymouth, MN, USA), with the media being changed every 2–3 days. Once the cells reached 95% confluency, they were washed and subcultured at a ratio of 25,000 cells/cm^2^ in new 100 mm cell culture dishes, which were marked as P1. Subsequent cultures were named P2, P3, P4, and so on. The P4 cells were used for the MSC differentiation experiments. The procedure followed in this study is summarized and depicted in Figure 2 below.

### 2.3. Cell Viability Assay

The cell viability was assessed using cellular 3-(4,5-dimethylthiazol-2-yl)-2,5-diphenyltetrazolium bromide (MTT) kits (Cayman Chemical, Ann Arbor, MI, USA). The cells were planted in 96-well black well culture plates containing a differentiation medium at a concentration of 5 × 10^4^ cells/100 μL. In the current study, the impact of treating MSCs with various concentrations of LPS (3.25, 6.25, 12.5, 25, 50, 100, and 200 μg/mL, Sigma-Aldrich) was analyzed during the culture period. The cells were exposed to different LPS treatments for 24 and 48 h in the dark, but the MTT viability assay was not performed for longer periods due to high cell density, which led to high absorbance readings that impeded the detection accuracy. DMEM with 10% MTT was added and incubated for 4 h, after which the culture medium was removed completely. The generated formazan was dissolved using 100 µL dimethyl sulfoxide (DMSO; Sigma-Aldrich) to create a purple color, and the plates were positioned on an orbital shaker (VWR) set at a low-speed setting for 5 min. The microplate reader (BioTek, Winooski, VT, USA) was used to measure the absorbance at 570 nm.

### 2.4. Osteogenic Differentiation

The cMSCs that were at P4 were put into 24-well plates at a density of 20,000 cells/cm^2^ for Alizarin Red S (AZ), alkaline phosphate (ALP), and Von Kossa (VK) staining, and in 6-well plates to measure the osteogenic gene regulation. These cells were cultured in basal media that contained DMEM, 10% FBS, 100 IU/mL penicillin, and 100 μg/mL streptomycin until they reached 90% confluency. Once they were confluent, they were treated with osteogenic media that contained DMEM with 10^−7^ M dexamethasone (DXA), 10 mM β-glycerophosphate, 50 μg/mL ascorbate, and 5% FBS to induce osteogenesis. Cells cultured in DMEM basal media with 10% FBS were used as a negative control. The old media was replaced with fresh media in the culture plate every 3 days. The cells were stained using AZ and VK for the detection of mineralization and using ALP to measure the osteogenic differentiation on days 7 and 14 of treatment.

### 2.5. Alizarin Red S Staining (AZ) and Mineral Deposit Quantification

In this study, the level of mineralization in chicken MSCs was measured using a technique called Alizarin Red S staining [4,18]. The cells were initially cultured in 24-well plates coated with gelatin and incubated in a growth medium until they reached full confluency. Next, the cells were exposed to LPS in an osteogenic differentiation medium for 7 and 14 days. On each day of staining, the cells were fixed using neutral buffered formalin and then stained with Alizarin Red S (Sigma-Aldrich, St. Louis, MO, USA). Mineralized nodules were identified as dark red spots, which were captured in 4X magnification using a microscope (Keyence BZ-X800, Keyence Corp., Itasca, IL, USA).

### 2.6. Alkaline Phosphatase Expression Assay

Alkaline phosphatase (ALP) expression was used in conjunction with the SIGMAFAST BCIP/NBT substrate (Sigma Aldrich, St. Louis, MO, USA) to identify osteoblast differentiation [4,18]. A successful differentiation of MSCs into osteoblasts is indicated by ALP activity [18]. When given BCIP/NBT as a substrate, differentiated osteoblasts were stained blue-violet while undifferentiated MSCs had a less intense color, which indicated weak alkaline phosphatase (ALP) activity. The cells were cleaned in PBS before being fixed in pre-cooled methanol for five minutes at −20 °C. After that, the sample was rinsed with distilled H2O before being incubated with dissolved SIGMAFAST BCIP/NBT substrate for 10 min at room temperature with steady agitation on a plate shaker. The cells were washed with distilled H2O after the stain solution was withdrawn, and images were taken right away using a microscope (Keyence BZ-X800, Keyence Corp., Itasca, IL, USA) while the cells were kept wet.

### 2.7. Von Kossa Staining

The cells in the culture plates were washed with PBS and fixed with 0.1% glutaraldehyde in PBS for 15 min at room temperature at different time points [4,18]. Then, the cells were washed with distilled water and exposed to 5% silver nitrate for 30 min. After washing the cells with distilled water and air-drying them, they were exposed to bright light until a black color appeared in the calcified areas. Images of the cell culture plates were taken using a microscope with a camera at 4x magnification (Keyence BZ-X800, Keyence Corp., Itasca, IL, USA).

### 2.8. Cellular ROS Assay

The intracellular ROS formation was evaluated using DCFDA/H2DCFDA Cellular ROS Assay Kits (ab113852, ABCAM, Waltham, MA, USA). The manufacturer’s instructions were followed to perform this assay at 1, 2.5, and 4 h after treatments.

### 2.9. Gene Expression

Cells plated in 6-well plates were harvested at 6, 12, 24, and 48 h to analyze the gene expression of bone formation and resorption markers using Quantitative Reverse Transcription Polymerase Chain Reaction (qRT-PCR). To detect the osteogenic differentiation of the cMSCs, thr mRNA expression of Runt-related transcription factor 2 (RUNX2) and bone morphogenetic protein (BMP2) was analyzed. The RNA was isolated from the cells using QIAzol lysis reagents, and the quantity of the RNA was determined using a Nano-Drop 1000 Spectrophotometer. The high-capacity cDNA reverse transcription kits were used to synthesize the cDNA from 2000 ng of total RNA. The mRNA expression was measured using qRT-PCR with primers designed using the Primer-BLAST program. The specificity of the primers was validated using PCR product sequencing. The qRT-PCR was performed in an Applied Biosystems StepOnePlus™ systems using iTaq™ Universal SYBR Green Supermix, with melting curve analysis and gel electrophoresis used to verify the primer quality. The qRT-PCR conditions were the same for all genes. These conditions included an initial denaturation at 95 °C for 10 min, followed by 40 cycles at 95 °C for 15 s, an annealing temperature for 20 s, and extending at 72 °C for 1 min. The primers used in this study are listed in Table 1.

### 2.10. Statistical Analysis

Statistical analysis was performed using ANOVA in the JMP^®^ Pro 16.0.0 software (SAS institute, Cary, NC, USA, 2023). A mean separation test was conducted using a Tukey test and *p* ≤ 0.05 was considered statistically significant among the groups.

## 3. Results

### 3.1. Cell Viability, Osteogenic Differentiation, and ROS Production

**Figure 3:**
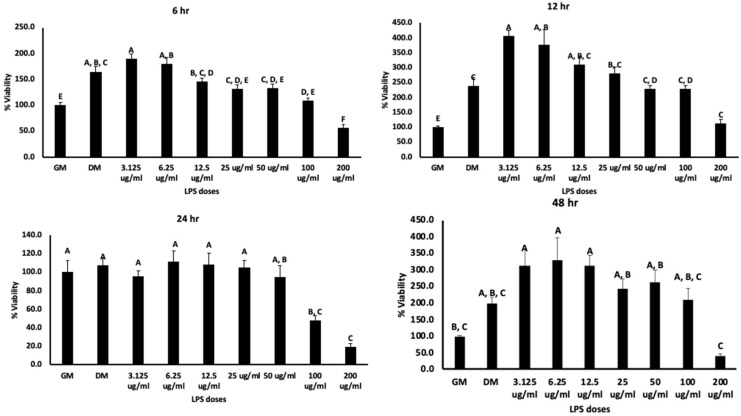
Represents the cell viability assay when MSCs were treated with LPS conducted at 6, 12, 24, and 48 h. GM and DM are negative and positive controls, respectively. Treatments with different letters indicate significant differences between treatments using Tukey’s HSD test, *p* < 0.05. Data shown include mean ± SEM of six individual replicates (*n* = 6).

**Figure 4:**
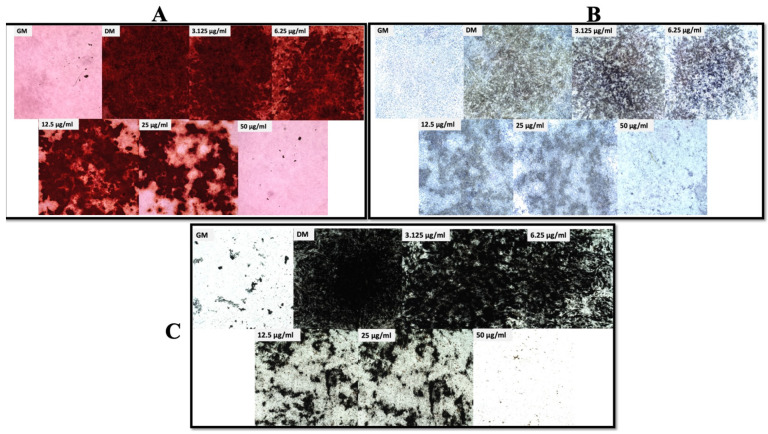
Represents the osteogenic differentiation staining at day 14, which revealed the decrease in differentiation (**A**,**C**) and alkaline phosphatase activity (**B**) with an increase in dose of lipopolysaccharides (LPS). GM and DM are negative and positive controls, respectively.

**Figure 5:**
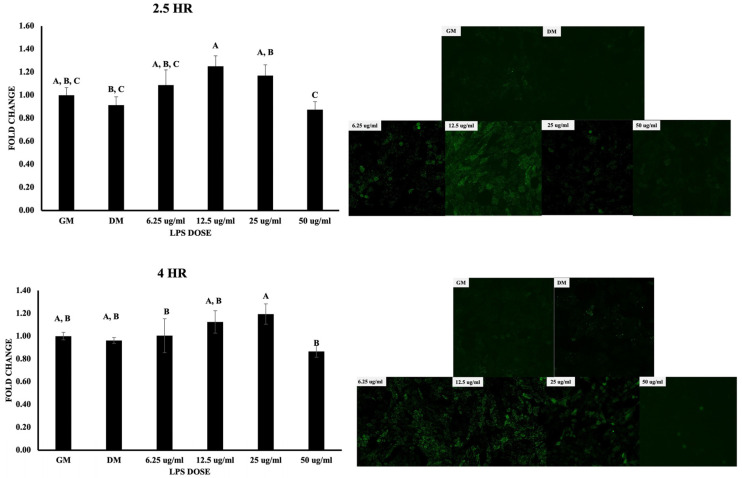
Represents the ROS production in chicken MSCs at 2.5 and 4 h of treatment. Treatments with different letters indicate significant differences between treatments using Tukey’s HSD test, *p* < 0.05. Data shown include mean ± SEM of five individual replicates (*n* = 5).

The LPS doses in this study were decided based on the results obtained using MTT assay (cell viability assay) (Figure 3). Chicken MSCs were treated with doses ranging from 3.125 to 200 μg/mL in an osteogenic medium for 6, 12, and 24 h. The trend in this assay was similar at all the time points. Precisely, when compared with growth media, the viability percentage for DM was nearly 165 and the viability for 3.125, 6.125, 12.5, 25, 50, 100, and 200 μg/mL doses of LPS at the 6 h time point was nearly 190, 180, 145, 131, 133, 108, and 56, respectively. Additionally, for the 12 h time point, the viability for the above doses was 239, 407, 377, 310, 278, 229, 228, and 112%, respectively, but these values at 24 h were 107.5, 96, 111, 108, 105, 95, 48, and 19%, respectively. By the end of 24 h and 48 h, 200 μg/mL showed significant reduction in cell viability by less than 25% and 50% (*p* < 0.05; Figure 3). Varying statistically significant differences were observed between the remaining doses but not much among 3.125, 6.25, 12.5, 25, and 50 μg/mL. Hence, the above doses were considered for the current study. Seeded mesenchymal stem cells were then subjected to osteogenic differentiation and stained with Alizarin Red, Von Kossa, and alkaline phosphatase. Using these staining methods, a decrease in osteogenic differentiation was observed from 3.125 μg/mL to 50 μg/mL on day 7 and day 14 (Figure 4). Furthermore, the relation between intracellular ROS and the osteogenic differentiation was evaluated using cellular DCFDA assay, and images are presented in Figure 5. The ROS response was observed at 2.5 h. 12.5 μg/mL showed a significant increase compared to DM and this was significantly lower for 50 μg/mL compared to 12.5 and 25 μg/mL (*p* < 0.05) but not at the higher doses (25 and 50 μg/mL). At 4 h of treatment with varying doses, observable changes are seen in Figure 5, showing that the ROS response was higher in 12.5 and 25 μg/mL but not with many significant differences in the quantified data. In contrast, there are significant differences for 12.5 μg/mL compared to 6.25 and 50 μg/mL.

**Figure 6:**
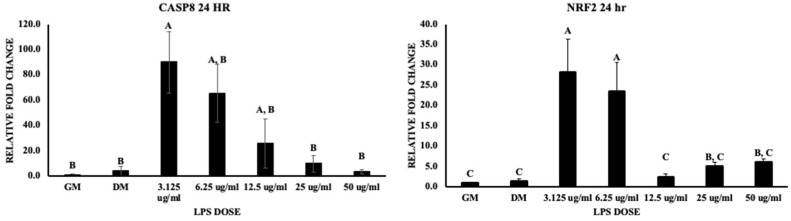
Represents the effect of LPS on chicken MSCs in CASP8 and NRF2 gene expression at 24 h of treatment. Treatments with different letters indicate significant differences between treatments using Tukey’s HSD test, *p* < 0.05. Data shown include mean ± SEM of three individual replicates (*n* = 3).

The mRNA expression of CASP8 (pro-apoptotic marker) and transcription factor Nrf2 (major regulator of antioxidant and cellular protective genes) was observed at 24 and 48 h from the addition of treatments. The data indicated a dose-dependent decrease in CASP8 gene expression at 24 h, but this was significantly (*p* value < 0.05) upregulated at 3.125 μg/mL (Figure 6). Furthermore, NRF2 was significantly upregulated by lower doses (3.125 and 6.25 μg/mL) at 24 h (*p* value < 0.05) but the controls and other treatments were not significantly, although there was an increase in gene expression in the treatments compared to the controls.

### 3.2. Inflammatory Pathways

**Figure 7:**
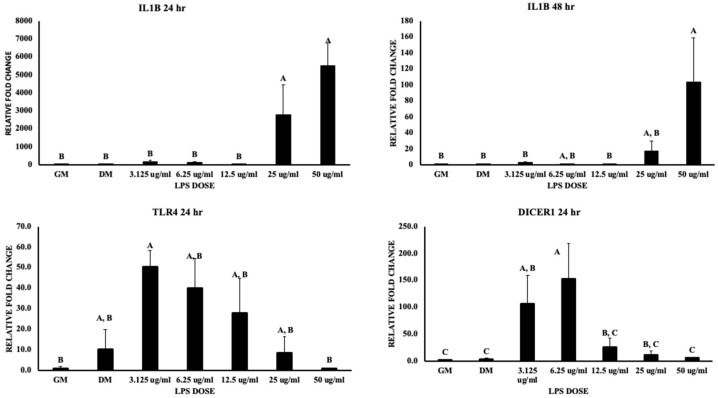
Represents the effect of LPS on chicken MSCs in terms of IL-1β, TLR4, and DICER1 gene expression at 24 h of treatment (48 h treatment for IL-1β). Treatments with different letters indicate significant differences between treatments using Tukey’s HSD test, *p* < 0.05. Data shown include mean ± SEM of three individual replicates (*n* = 3).

Pro-inflammatory cytokine IL-1β (interleukin 1β) was upregulated with a 3000- and 6000-fold increase in the gene expression for the 25 and 50 μg/mL doses, respectively, at 24 h (*p* value < 0.05), and a greater than 100-fold increase at 48 h (*p* value < 0.05) (Figure 7). Moreover, the toll-like receptor 4 (TLR4) signal pathway plays a key role in triggering the innate immune response and this is responsible for acute and chronic inflammatory disorders due to the activation of early phase nuclear factor-κB (NF-κB), which further leads to the production of inflammatory cytokines. The current study showed a significant upregulation (nearly 50-fold increase) of TLR4 at 3.125 μg/mL compared to 50 μg/mL of LPS (*p* value < 0.05) (Figure 7). Additionally, NF-κB expression was not significant. Dicer1, an endoribonuclease that regulates miRNA maturation, showed a greater than 100-fold increase at 3.125 μg/mL and a nearly 150-fold increase after 24 h (*p* value < 0.05) (Figure 7).

### 3.3. Wnt Signaling and Bone Homeostasis

**Figure 8:**
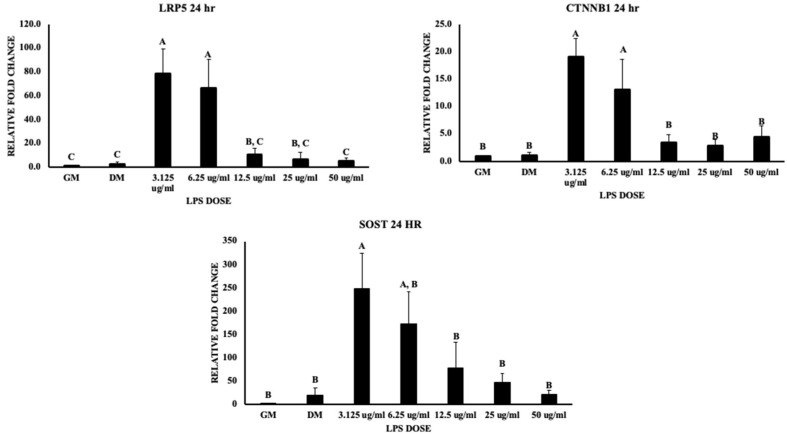
Represents the effect of LPS on chicken MSCs in LRP5, CTNNB1, and SOST gene expression at 24 h of treatment. Treatments with different letters indicate significant differences between treatments using Tukey’s HSD test, *p* < 0.05. Data shown include mean ± SEM of three individual replicates (*n* = 3).

**Figure 9:**
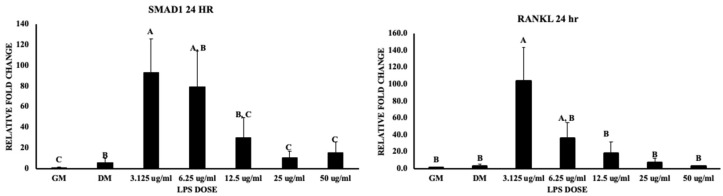
Represents the effect of LPS on chicken MSCs in SMAD1 and RANKL gene expression at 24 h of treatment. Treatments with different letters indicate significant differences between treatments using Tukey’s HSD test, *p* < 0.05. Data shown include mean ± SEM of three individual replicates (*n* = 3).

The canonical Wnt signaling pathway is associated with early and ultimate osteoblast differentiation stages, but a physiological balance should be met to positively promote osteoblastogenesis. Dysregulated elevation of LRP5 and Wnt signaling is associated with more bone synthesis with normal resorption, but deficiency leads to the inhibition of canonical Wnt signaling. The lipoprotein receptor protein (LRP5) gene expression was significantly upregulated nearly 80-fold and 70-fold at 3.125 and 6.25 μg/mL, respectively (*p* value < 0.05). Similarly, this study showed that beta-catenin at 24 h was significantly upregulated nearly 20-fold and 13-fold at 3.125 and 6.25 μg/mL, respectively (*p* value < 0.05) (Figure 8). In contrast, sclerostin (SOST), which is an inhibitor of the Wnt signaling pathway, was significantly upregulated nearly 250-fold with 3.125 μg/mL of LPS, with a 180-fold increase in gene expression with 6.25 μg/mL, followed by a decrease in expression with increasing LPS doses (*p* value < 0.05) (Figure 8). In view of osteogenesis, RUNX2 (Runt-related transcription factor) is an upstream transcription factor (master regulator of osteogenesis) involved in osteoblast differentiation. This gene expression was not significant in MSCs subjected to LPS doses. BMP2 is upstream to RUNX2 and is a potent osteogenic agent responsible for the maturation of mesenchymal osteoprogenitor cells into osteoblasts. BMP signaling is responsible for RUNX2 transcriptional activity. The treatments did not show significant differences for all LPS doses at 24 and 48 h (*p* value = 0.3580, 0.5316). BMP’s osteogenic role is activating SMAD1 and mediating RUNX2 expression. This study showed an upregulation in SMAD1 signaling at 3.125 μg/mL LPS (nearly 70-fold) (*p* value < 0.05) and an 80-fold increase with 6.25 μg/mL (Figure 9). Osteoprotegerin (OPG), which is a decoy receptor for RANKL to inhibit osteoclastogenesis and osteoclast activation, was not significant (*p* value = 0.0615). In contrast, the RANKL expression was significantly upregulated at 3.125 (100-fold increase) and 6.25 μg/mL (40-fold) at 24 h (*p* value < 0.05) but not significant at 48 h of treatment (*p* value = 0.7545) (Figure 9), which indicates a higher expression of RANKL favoring osteoclastogenesis. These results indicate the potential roles of LPS in bone formation and bone resorption by regulating Wnt signaling and osteoclastogenesis; LPS potentially reduces osteogenic differentiation and increases osteoclastogenesis.

## 4. Discussion

The interaction between the immune system and bone development has been confirmed by several researchers and by previous studies, which provided a great insight into developing new mitigating strategies against inflammatory bone homeostasis dysregulation [4,19]. Surprisingly, few to none of the studies elucidated the dose-dependent effect of bacterial LPS on chicken MSCs or human-derived MSCs, which would help in alleviating the issues associated with this pathogenic factor at different doses. In this study, dose-dependent variation in osteogenic differentiation was observed, and the data revealed that the immune and differentiation responses greatly varied with the nidus of the inflammatory stimulus. Higher doses in this study (50 μg/mL) of LPS affected the osteogenic differentiation greatly with mere mineralization on days 7 and 14 without affecting the cell viability. Moreover, there is decreased osteogenic potential with the subsequent lowering of LPS doses. The cell viability was not affected by the LPS dosing from 3.125 μg/mL to 50 μg/mL, indicating its potential to be a valuable tool to conduct, for example, inflammation research, and the current findings agree with a study conducted on human bone-marrow-derived MSCs. Osteogenic differentiation staining revealed that higher doses of LPS affected MSCs, lowering their osteogenic potential, but showed a dose-dependent increase in mineralization with decreasing doses of LPS. LPS at 10 μg/mL in human periodontal ligament stem cells impaired the osteogenic differentiation ability but not with human bone marrow MSCs [13]. This study revealed the underlying mechanisms behind the effect of LPS on osteogenic differentiation when utilized at various doses.

### 4.1. Inflammatory Pathways

The current study showed the increased gene expression of IL-1β with an increase in LPS dose, possibly due to the dysregulation of Dicer1 even in the presence of higher doses of LPS, which leads to accumulation of double-stranded RNA (dsRNA) and triggers the formation of IL-1β from pro- IL-1β. In contrast, lower doses of LPS have shown an increased gene expression, which could have provided some protective effect preventing dsRNA accumulation, further preventing IL-1β formation [16,20,21]. This mechanism was reported to play a key role in osteogenesis and showed dysregulation in several human diseases, thus leading to a break in bone homeostasis [16,22,23]. TLR4, a transmembrane receptor, was upregulated in lower doses but not at higher doses, indicating that the effect of high doses might be transient and follow a different pathway, like Dicer dysregulation leading to elevated gene expression with higher LPS doses. Furthermore, literature related to the transient effect of LPS was not found, but a study on rats showed that the liver mRNA expression at higher and lower LPS doses was elevated [24]. Additionally, the mRNA expression of pro-inflammatory cytokines was limited in the muscle compared to the liver, spleen, and kidneys in rats injected with LPS intraperitoneally, indicating that TLR4 activation varies based on the type of tissue and origin of cells, although dose-dependent relation was also limited [25]. The data from the current study revealed the role of Dicer1 dysregulation in the higher gene expression of pro-inflammatory cytokine (IL-1β), but a contrasting observation with respect to TLR4 mRNA expression, which reveals the effect of LPS at higher doses would be transient but dysregulates Dicer1 signaling.

Some studies reported an increase in superoxide production in mitochondria along with a decrease in osteogenic differentiation, which is due to the activation of inflammasome and IL-1β release [26]. In contrast, the current study found that the ROS response increased at 12.5 μg/mL but not at higher doses or other doses at 2.5 and 4 h after treatment. This indicates that the ROS generation at higher doses would be momentary and greater amounts of IL-1β would be released, which was observed in the current data, along with the release of this cytokine in relatively very scarce amounts even though there was ROS generation at 3.125, 6.25, and 12.5 μg/mL. These findings are different from the other studies because those studies found ROS generation would activate inflammasome and induce NF-κB pathway activation. This hold true in this study since the NF-κB along with the NLRP inflammasome gene expression was insignificant, which revealed that the Dicer1 pathway takes the upper hand when compared with other pathways, leading to higher IL-1β gene expression and thus a reduction in the osteogenic differentiation of MSCs. On the other hand, the findings in this study could be because the ROS assay was analyzed at 2.5 and 4 h, but IL-1β was observed at 24 and 48 h [27,28]. The microenvironment surrounding the MSCs can alter the physiology and osteogenic differentiation, like regulating the polarization of macrophages and enhanced immunosuppressive effects, but this is still undeciphered [29,30]. Evidence shows that upregulation of pro-inflammatory cytokines enhances the cell plasticity, along with immune regulatory characteristics, but this depends on the concentration of cytokines [31]. The current study found the upregulation of IL-1β, which may affect the microenvironment, thus affecting the osteogenic differentiation by downregulating SMAD1, which agrees with the previous statement.

### 4.2. The Wnt Signaling Pathway

**Figure 10:**
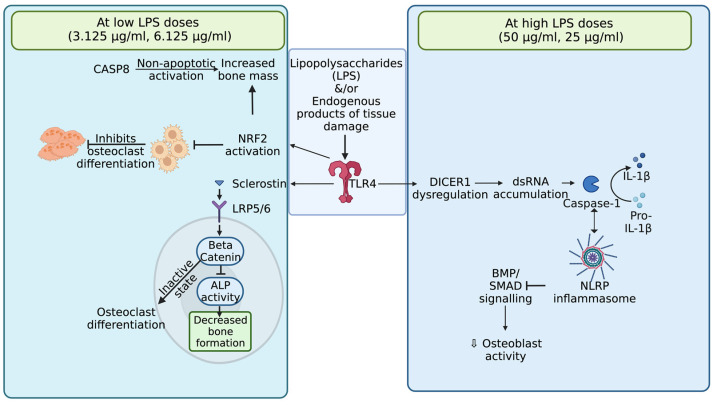
Summarizes the possible pathways affecting osteogenic differentiation in the current study at lower (3.125 μg/mL and 6.25 μg/mL) and higher (25 μg/mL and 50 μg/mL) doses.

Wnt signaling is an important pathway in tissue homeostasis and abnormal signaling leads to degenerative diseases. Furthermore, the activation of this pathway, stimulated by pathogens, reduces several molecular inflammatory processes [32]. The current study showed that the Wnt signaling was also compromised, which was observed with LRP5 and beta-catenin downregulation when increasing the LPS doses in all treatments. Studies have shown that Wnt/beta-catenin cascade activation results in mesenchymal precursors to osteoblast differentiation, where inhibition leads to impaired growth [33,34,35]. At lower LPS doses like 3.125, 6.25, and 12.5 μg/mL, there was upregulation of beta-catenin and LRP5 receptor gene expression, which reveals that lower doses of LPS should be able to trigger osteogenic differentiation, but this is in contrast to findings from other studies [14,22,32]. In contrast, the data showed that the sclerostin mRNA expression was upregulated with lower LPS doses (3.125, 6.25, 12.5 μg/mL), which indicates its inhibitory role in the Wnt signaling pathway by creating a negative feedback loop of osteogenesis by binding to the LRP5 receptor. This reveals that the trigger from LRP5 and beta-catenin activation that supports osteogenic differentiation will be inhibited with a significantly higher gene expression of sclerostin. Furthermore, this leads to decreased mineralization compared to the solely differentiation-media-treated MSCs conclusively being affected in their proliferation and differentiation [34,35]. Finally, the current study shows that several pathways, which are illustrated in Figure 10 are synergistic and linked to each other in causing the decrease in osteogenic potential, but this varies with the dose of LPS. This evidently indicates the role of the Wnt signaling pathway in regulating the bone formation, along with the role of sclerostin in countering the Wnt pathway at lower doses of LPS, coupled with DICER1 dysregulation affecting bone formation at higher doses of LPS.

### 4.3. Bone Formation and Resorption

Bone formation and remodeling are key processes involved in aiding cells like MSCs to adapt to their microenvironment and external stimuli. This homeostasis in bone remodeling is maintained via bone resorption by osteoclasts and bone formation by osteoblasts, which are maintained by the RANKL, SMAD1, DICER1, and Wnt signaling pathways, as described in the current study. SMAD is an intracellular signaling protein which phosphorylates transforming growth factor(TGF)-β and bone morphogenic proteins (BMPs); these act on transcription factors like RUNX2 and Osx, which are crucial in the osteogenic differentiation of MSCs [18,22,36]. The current study has shown a higher mRNA expression of SMAD1 with lower doses of LPS (3.125, 6.25, 12.5 μg/mL), indicating the increase in bone formation, but RUNX2, which is key transcription factor, was insignificant: this shows the incongruity of this factor with the intracellular signaling molecule (SMAD1). These findings indicates that, even with an increase in the gene expression of SMAD1, RUNX2 recruitment was not in synchrony, thus leading to relatively lesser mineralization compared to the positive control (DM). Furthermore, some studies proved the relation between SMAD and RUNX2 along the higher expression of the latter inducing osteogenic potential [37]. The current study revealed that the SMAD1 mRNA expression does not necessarily lead to the transcription of downstream regulators like RUNX2. Additionally, this also might be linked to Wnt signaling and other inflammatory triggers in promoting osteogenesis.

Secondly, the other important component of remodeling is bone resorption. RANKL (receptor activator of nuclear factor-κB) is an important gene expression marker and primary regulator involved in bone resorption [22,38]. The gene expression of RANKL was significantly upregulated with lower doses (3.125, and 6.25 μg/mL), indicating the potential activation of resorbing cells at these doses, which would represent a turnover with respect to bone homeostasis. This statement was supported by the gene expression of SMAD1, which was shown in the preceding paragraph. This increased gene expression of SMAD1 and RANKL represents a heightened scope for bone formation and resorption, respectively, but this trend was similar with other doses of LPS, with gene expression weakening with an increase in LPS. In contrast to the statements supporting the ability of RANKL to resorb a bone, an anabolic effect was observed in vesicular RANK and RANKL-binding peptides but less in RANKL-deficient osteoblasts [39]. The data from the current study revealed the RANKL is lenient toward resorption and would also support bone formation (an anabolic effect) with lower doses of LPS (3.125, and 6.25 μg/mL) compared to the others.

## 5. Conclusions

MSCs, which have the potential to differentiate into osteoblasts, can be an excellent model to provide valuable insights into resolving the issues with bone-related diseases beyond the concerns in the poultry industry, especially bacterial chondronecrosis and osteomyelitis. This study suggests that these pathways discussed in the current study are all linked to each other in the MSCs’ microenvironment, which significantly affects osteogenic differentiation. Additionally, the varied response with different doses of LPS showed the synchronized mechanisms in altering bone homeostasis. Furthermore, these varied at higher and lower levels of LPS. Precisely, lower doses of LPS affected bone homeostasis through the inhibition of Wnt signaling from sclerostin, and higher levels of LPS affected the IL-1β pathway, which could be related to Dicer1 dysregulation. Although there are limitations in deciphering some issues, the data obtained in this study have provided potential therapeutic targets at various doses of LPS, which can be related to inflammatory bone diseases in several fields, including the poultry industry.

## Figures and Tables

**Table 1:** Represents all the primers used in this study with their respective gene names, forward primers, reverse primers, and accession numbers.

Gene Name	Accession Number	F Primer Sequence	R Primer Sequence
BMP 2	NM_001398170.1	CCCCTACATGTTGGACCTC	CCCACTTGTTTCTGGCAGT
RUNX2	NM_204128.2	GTGGCCAGATTCAATGACCT	CCATCCACCGTCACCTTTAT
CTSK (Cathepsin K)	NM_204971.3	AGTCTGCCCTCCTTCCAGTT	CTTGATGATCCAGTGCTTGG
TNFSF11 (RANKL)	NM_001083361.2	GTCCAGCGTATTCTGGGAAA	GCAAAAGGTTGCTTCTCTGG
LRP5	NM_001012897.2	GGTGCCCCCTTATATGACAG	GATCAGTAGCTGGGGATGGA
TLR4	NM_001030693.2	ACTCTTGGGGTGCTGCTG	TGTCCTGTGCATCTGAAAG
b-catenin (CTNNB1)	NM_205081.3	AGGGTGCTGAAGGTGTTGTC	GCTGGCTTGGATCTGTAAGG
Dicer1 (ribonuclease 3)	NM_001040465.2	GACCTGACCAATCTCAACCAG	TTTGCCTTCCTCTTCTCAGC
NFKB2	NM_204413.2	CCACGTCACCAAGAAGAACA	GGTCCATCACCTTCTTCAGC
NFE2L2 (NRF-2)	NM_205117.2	ATGCAGCTCTTGGCAGAAG	CTGGGTGGCTGAGTTTGATT
OPG	NM_001033641.1	ACGCTTGTGCTCTTGGACAT	CAGCGTAGTACTGGTCTGGG
IL-1B	XM_015297469.2	TGCCTGCAGAAGAAGCCTCG	GACGGGCTCAAAAACCTCCT
SOST	XM_004948551	ATCCCACCTCCTGCCCAACTCCATC	GGTTCGGTTTGCTGCTCCTGGCTC
SMAD1	XM_040698719.1	GTTTTGTAAAGGGTTGGGGAGC	AATGCAGGAGCTTGGGACCTTA
Beta-actin	NM_205518.2	CAACACAGTGCTGTCTGGTGGTA	ATCGTACTCCTGCTTGCTGATCC

## Data Availability

The authors of this article are committed to providing the raw data supporting their conclusions without any hesitation or reservation.
